# Attention-deficit/hyperactivity disorder and brain metabolites from proton magnetic resonance spectroscopy: a systematic review and meta-analysis protocol

**DOI:** 10.47626/2237-6089-2019-0111

**Published:** 2021-02-26

**Authors:** Marcos Vinícius Vidor, Alana Castro Panzenhagen, Alexandre Ribeiro Martins, Renata Basso Cupertino, Cibele Edom Bandeira, Luis Augusto Rohde, Diego Luiz Rovaris, Claiton Henrique Dotto Bau, Eugênio Horácio Grevet

**Affiliations:** 1 Programa de Transtornos de Déficit de Atenção/Hiperatividade em Adultos Centro de Pesquisa Clínica Hospital de Clínicas de Porto Alegre Porto AlegreRS Brazil Programa de Transtornos de Déficit de Atenção/Hiperatividade em Adultos (ProDAH-A), Centro de Pesquisa Clínica (CPC), Hospital de Clínicas de Porto Alegre (HCPA), Porto Alegre , RS , Brazil .; 2 Programa de Pós-Graduação em Psiquiatria e Ciências do Comportamento Universidade Federal do Rio Grande do Sul Porto AlegreRS Brazil Programa de Pós-Graduação em Psiquiatria e Ciências do Comportamento , Universidade Federal do Rio Grande do Sul (UFRGS), Porto Alegre , RS , Brazil .; 3 Programa de Pós-Graduação em Ciências Biológicas: Bioquímica UFRGS Porto AlegreRS Brazil Programa de Pós-Graduação em Ciências Biológicas: Bioquímica , UFRGS , Porto Alegre , RS , Brazil .; 4 Serdil – Clínica de Radiologia e Diagnóstico de Imagem Porto AlegreRS Brazil Serdil – Clínica de Radiologia e Diagnóstico de Imagem , Porto Alegre , RS , Brazil .; 5 Department of Psychiatry University of Vermont BurlingtonVT USA Department of Psychiatry , University of Vermont , Burlington . VT , USA .; 6 Departamento de Genética Instituto de Biociências UFRGS Porto AlegreRS Brazil Departamento de Genética , Instituto de Biociências , UFRGS , Porto Alegre , RS , Brazil .; 7 Instituto Nacional de Psiquiatria do Desenvolvimento Conselho Nacional de Desenvolvimento Científico e Tecnológico Porto AlegreRS Brazil Instituto Nacional de Psiquiatria do Desenvolvimento (INPD), Conselho Nacional de Desenvolvimento Científico e Tecnológico (CNPq), Porto Alegre , RS , Brazil .; 8 Instituto de Ciências Biomédicas Departamento de Fisiologia e Biofísica Universidade de São Paulo São PauloSP Brazil Instituto de Ciências Biomédicas , Departamento de Fisiologia e Biofísica , Universidade de São Paulo (USP), São Paulo , SP , Brazil .

**Keywords:** MRS, spectroscopy, ADHD, meta-analysis, protocol

## Abstract

Despite major advances in the study of the brain, investigations on neurochemistry in vivo still lack the solid ground of more established methods, such as structural and functional magnetic resonance imaging. Proton magnetic resonance spectroscopy (MRS) is a technique that might potentially fill in this gap. Nevertheless, studies using this approach feature great methodological heterogeneity, such as varying voxel of choice, differences on emphasized metabolites, and absence of a standardized unit. In this study, we present a methodology for creating a systematic review and meta-analysis for this kind of scientific evidence using the prototypical case of attention-deficit/hyperactivity disorder. **Systematic review registration:** International Prospective Register of Systematic Reviews (PROSPERO), CRD42018112418.

## Introduction

Attention-deficit/hyperactivity disorder (ADHD) is a neuropsychiatric condition characterized by symptoms of inattention, hyperactivity, and impulsivity. ^1^ It is a prevalent disorder among children and adolescents that often persists into adulthood. ^2 , 3^ ADHD is also highly associated with comorbid psychiatric disorders ^3^ and negative outcomes, such as poor quality of life, ^4^ unemployment, ^5^ and increased mortality. ^6^ The economic burden associated with ADHD in the U.S. alone is estimated to be US$67-116B yearly due to lack of productivity. ^5^ Even so, key aspects of the neural basis of the disorder remain to be unmasked. ^7 , 8^

Since the 1990s, major advances in technology have made it possible to better understand brain diseases through the study of the brain *in vivo* . ^9^ Functional magnetic resonance imaging studies, for instance, have shown the role of frontoparietal and default mode network systems on ADHD. ^10^ Nevertheless, some areas of neuroscience, such as neurochemistry, have shown a more modest progress, still majorly relying on either *post-mortem* or more indirect approaches. ^11^ Given this framework, proton magnetic resonance spectroscopy (MRS) appears to be a link to partially fill in this gap and promote an upgraded landscape on the study of the biochemistry of living tissues. ^12 - 14^

MRS is a technique based on the concept of chemical shift, which describes how electronic shielding of an atomic nucleus embedded in a more complex chemical compound – i.e., a nucleus not free – changes its resonance frequency. ^15^ This allows us to establish different fingerprints to different molecules based on specific resonance frequencies of protons and estimate their amount on a certain pre-specified volume of interest. Following this principle, some molecules are classically found on MRS studies of neural tissue, such as n-acetylaspartate, creatine, choline, glutamate, myo-inositol, and lactate. ^16^

The first MRS studies of the brain of living organisms were performed in the 1980s, ^17 , 18^ whereas ADHD spectroscopy began in the early 2000s ^19 , 20^ ; the technique has been progressively more used since then. Nonetheless, the increasing amount of data available regarding this approach has revealed to be somehow problematic for those willing to summarize the knowledge generated, especially on the grounds of methodological heterogeneity within studies focusing on the same disease. There are at least three levels of variation among MRS studies. First of all, the voxels studied greatly vary for each design. Secondly, not all the studies identify the same metabolites. Finally, there is no standard unit for the results. In an MRS chart, one axis indicates frequency, and the other identifies an arbitrary unit influenced by many factors such as voxel size and radiofrequency coil sensitivity. ^21^ In addition to that, some authors use pure metabolite quantifications, whilst others prefer ratios of metabolites, usually using creatine as a baseline. ^22 , 23^

The present study aims to delineate a method for grouping the data of MRS studies of patients with ADHD. More specifically, we describe a methodology for creating a systematic review and meta-analysis for this kind of scientific evidence. As a meta-analysis combines statistical findings of different studies addressing the same scientific problem to extract a single result, it seems to qualify as an appropriate method to tackle the difficulties found in the field. ^24^ Our main goal in this protocol is to describe a methodology capable of circumventing the aforementioned setbacks of merging spectroscopy results. Hopefully, with this work, we will be able to establish whether there are consistent differences among brain metabolites of patients with ADHD and those of healthy controls, and additionally, in doing so, to evaluate the reliability of the MRS approach as performed thus far.

## Methods

This protocol has already been registered on the International Prospective Register of Systematic Reviews (PROSPERO; https://www.crd.york.ac.uk/prospero) under the title “Magnetic resonance spectroscopy on attention deficit/hyperactivity disorder: systematic review and meta-analysis” and ID number CRD42018112418. The outline of our protocol is in accordance with the 17-item checklist of the Preferred Reporting Items for Systematic Reviews and Meta-Analyses for Protocols 2015 (PRISMA-P 2015). ^25 , 26^

### Search on databases

We are going to look for studies that measure brain metabolites through MRS in patients with ADHD and healthy controls. Using specific keywords previously selected ( Table 1 ), we are going to perform searches on the following databases: Embase, Google Scholar, PubMed, ScienceDirect, SciELO, Scopus, and Web of Science. We also plan on looking for unpublished works on ERIC, the International Clinical Trials Registry Platform (WHO ICTRP), CINAHL Database, and ProQuest Dissertations & Theses Global, using the keywords “ADHD” and/or “Attention-deficit hyperactivity disorder” for the condition, and “MRS,” “MR spectroscopy,” and/or simply “spectroscopy” for the technique. No restriction will be imposed on language or year of publication. All entries will be recorded in a comprehensive list. Duplicates will be removed and the remainder records will form the final list for eligibility evaluation. Once the studies are selected, we are going to look for further potential candidate articles in the reference lists of all the studies included ( Table 1 ).

**Table 1:** Databases and keywords

Database	Keywords
Embase	‘attention deficit disorder’ and ‘nuclear magnetic resonance’ and ‘spectroscopy’:ab,ti
Google Scholar	ADHD Attention-deficit hyperactivity disorder Nuclear magnetic resonance spectroscopy NMR spectroscopy MRS MR spectroscopy
PubMed	(ADHD[all fields] OR Attention-deficit hyperactivity disorder[all fields]) AND (Nuclear magnetic resonance spectroscopy[all fields] OR NMR spectroscopy[all fields] OR MRS[all fields] OR MR spectroscopy[all fields])
ScienceDirect	tak ((ADHD OR “Attention-Deficit/Hyperactivity Disorder”) AND (“Nuclear magnetic resonance spectroscopy” OR “NMR spectroscopy” OR MRS OR “MR spectroscopy”))
SciELO	(Nuclear magnetic resonance spectroscopy) OR (NMR spectroscopy) OR (MRS) OR (MR spectroscopy) AND (ADHD) OR (Attention-deficit hyperactivity disorder)
Scopus	TITLE-ABS-KEY ( ( adhd OR “Attention-deficit hyperactivity disorder” ) AND ( “Nuclear magnetic resonance spectroscopy” OR “NMR spectroscopy” OR mrs OR “MR spectroscopy” ) )
Web of Science	TS = (ADHD OR Attention-deficit hyperactivity disorder) AND TS = (Nuclear magnetic resonance spectroscopy OR NMR spectroscopy OR MRS OR MR spectroscopy)

### Study identification and selection

Two authors will independently analyze the whole list of studies to assess eligibility. Their evaluation will be matched for each entry and divergences (through percentage and Kappa statistics: κ ≡ (po – pe)/(1-pe) = 1 – (1-po)/(1-pe), where *po* is the relative observed agreement among raters (accuracy), and *pe* is the hypothetical probability of chance agreement. Whenever divergences are found, the articles will be finally assessed by a third author. In a first approach, studies not related to our investigation will be excluded on the basis of title and abstract. All remaining entries will be subjected to full-text reading, when other studies might be ruled out.

The following inclusion criteria will be considered: 1) all studies must contain at least one group with patients with ADHD and one group with healthy controls; 2) all studies must contain original proton MRS data on brain metabolites. When more than one diagnosis is considered, the studies will be included if there is a group comprised of patients with ADHD only. Cases will be defined as individuals diagnosed with ADHD according to the Diagnostic and Statistical Manual of Mental Disorders, 3rd edition (DSM-III) (attention deficit disorder [ADD]), DSM-III-R, DSM-IV, DSM-IV-TR, or DSM-5 through either professional direct evaluation or screening tools, for which the diagnostic value relies on the evaluation of the authors of each study. There will be no age, sex, or ethnicity restrictions. Controls will be defined as individuals verified as not having the condition through either professional direct evaluation or screening tools also based on DSM-III (ADD), DSM-III-R, DSM-IV, DSM-IV-TR, or DSM-5 criteria. No screening for other psychiatric conditions will be required, neither for cases nor for controls; this information will nonetheless be used as a quality criterion to be evaluated in our bias assessment, as described hereinafter.

### Study characteristics to be extracted

Extraction of data from each study will retrieve the following characteristics: year of publication, sample size of each group, male-to-female ratio in each group, aimed population (children/adolescents or adults), mean age of each group, regions of the brain studied, metabolites measured, and main results. The extraction will be performed by two authors, and divergences will be addressed by a third author. General principles of extraction followed the Cochrane Handbook for Systematic Reviews of Interventions. ^27^

Data directly related to the meta-analysis – i.e., mean metabolite measurement, standard deviation (or standard error), and sample size for each group (cases and controls) – will be recorded on independent worksheets, one for each brain region selected. The preferable sources are tables and written data on the article’s full text. When the information is not detected on these formats, graph estimation using a digital ruler ^28 , 29^ will be performed. In case the previous methods are not available in the study or supplemental material, an email requesting the information will be dispatched for the correspondence address indicated in the authors’ section of the article. If there is no response in two months, the data will be deemed as missing. Two authors will collect the data independently, and disparities will be corrected by discussion consulting the original source.

### Selection of brain regions for meta-analysis

Considering the heterogeneity of voxel choice among studies, it is important to delineate our approach on grouping data for the meta-analysis. While we anticipate that some degree of subjectivity will be unavoidable, we expect to proceed in a roughly systematic manner, as follows: on a first level, if enough studies are available, we will group data that target specifically the same regions (e.g., left dorsolateral prefrontal cortex); if not enough studies are available, we will combine more comprehensive data, but still on areas spatially related (e.g., left dorsolateral prefrontal cortex and left ventromedial prefrontal cortex); lastly, if the previous methods are not feasible, we intend to incorporate data from similar structures, disregarding laterality.

### Bias assessment

Risk of bias will be assessed using the Newcastle-Ottawa Scale (NOS) ^30^ in its case-control study format. The NOS is a quality scale composed of nine items grouped within three sections: selection, comparability, and exposure. Selection bias is assessed by four questions regarding definition of cases, representativeness of cases, definition of controls, and selection of controls. Quality of comparability between cases and controls is evaluated by a sole topic divided in two items that estimates how well controlled the healthy control group was. Finally, excellence on exposure measures is assessed by three items focusing on quality of records, methods of ascertainment for cases and controls, and non-response rate. The NOS provides a scale from 0 to 9, one point for each of the items mentioned, the final score being related to the quality of the study in a direct fashion. Two authors will perform the NOS evaluation for each study, and the disparities on matched results will be discussed among the evaluators on the basis of the methodological description of the studies. Publication bias will be assessed using funnel plots and Egger’s regression test.

### Data analysis

#### Meta-analysis

Studies will be grouped according to the aforementioned criteria and a meta-analysis will be performed for each metabolite of each region that meets a minimum of three values coming from at least three different studies. In articles with more than one ADHD group available (e.g., “treatment naïve ADHD group” and “on stimulants ADHD group”) in a way that the data cannot be coupled, all ADHD groups will be matched against the control group, with the sample size of the healthy control group divided by the number of ADHD groups rounded down. As for the example given, if both a “treatment naïve group” and an “on stimulants group” are present, both will be fully used as case groups, but matched against a control group divided by two (the number of case groups that meets the eligibility criteria), considering that the control group would be otherwise counted twice. Additionally, when combining different data from the same study (e.g., disregarding lateralization in a study with data from both sides of the brain, hence using two different sets of data from the same study, one for each side), both cases and controls will be divided by two and rounded down. These steps aim to avoid overestimation of sample sizes and both may be applied to the same work simultaneously if needed.

Standardized mean differences obtained through Hedge’s G method with random effects will be employed to determine pooled effect sizes. Significance will be established by a Z-test. Inverse variance will be used to determine individual study weights. Studies will also be assessed on their heterogeneity using the χ ^2^ and I ^2^ tests, considering p ≤ 0.01 as statistically significant. Low, moderate, and high heterogeneity values will be assumed from I ^2^ values of 25, 50, and 75%, respectively. ^31^ In light of the fact that the possible use of effect sizes from the same study would artificially decrease heterogeneity, we will employ a three-level analysis adopting “study” as an extra random variable. The R package *meta* will be used to assess standardized mean differences and heterogeneity values as well as to generate forest plots. ^32^

#### Sensitivity of analysis

We will perform sensitivity analyses in order to evaluate methodological disparities that might account for effect size differences. First, we intend to use the jackknife method to assess how dissonant studies might be affecting the outcome, excluding one result at a time from each meta-analysis; second, we will execute the analyses again after excluding studies with high risk of bias or rated as “unclear risk of bias” through a score related to our bias assessment from the NOS, as previously described, excluding studies rated as lower than one standard deviation from the mean (bootstrapping will be used to acquire normal distributions and set the thresholds in case the data is non-parametric); and third, we will divide the groups by age, gender, and field strength of the machinery, when enough data is available. Finally, we foresee an additional analysis including only published works to be performed as well.

## Discussion

The MRS seems to be a useful technique for in vivo brain investigations, especially when considering the limited availability of methods for studying neurochemistry in living neural tissue. The main limitation of this approach is that its use is only possible when high concentrations of metabolites are available (in the millimolar range), once the magnetic resonance method is poorly sensitive. Also, on a practical level, there is a significant spectral overlap among many compounds, making its discrimination a particular challenge. ^15^ Even so, it is quite puzzling that MRS is not more often used as a way of investigation. Some reasons for that might be speculated. First, the meaning of MRS results might still not be intuitively grasped on a physiological level – not in a degree that most scientists feel comfortable to rely on. In this sense, it is worth mentioning that more established methods, such as structural and functional magnetic resonance imaging, although probably to a different degree, can also fall in similar pitfalls. ^33 - 35^ Apart from that, MRS endures serious heterogeneity among studies, such as varying voxel of choice, occasional disparities on emphasized metabolites, and the absence of a standardized unit of measurement, all of which undermine its long-term technical appraisal.

In addition to undermining our interpretation of the data, the diversity observed in the use of this technology thus far is a particular obstacle for developing a protocol that aims to combine multiple results in one coherent statistical value. In previous meta-analyses of MRS data, different approaches were employed to address these problems. Concerning pooling brain regions, for instance, we observed that, while some studies do not detail their methods on this point, options included disregarding the brain area of source ^36^ – reasonable for specific neurological issues when functionality is not a primary concern for study purposes –; pre-specifying brain regions to be studied as inclusion criteria ^37^ ; choosing a preferable brain side of source when information from both hemispheres is available ^38^ ; and stratifying the data in brain lobes. ^39^ Meta-analyses also diverge on how to address metabolite measures. While most works screen for all the main proton MRS molecules, ^36 , 37 , 39 - 41^ some choose to focus on specific frequency ranges. ^38^ We chose to preliminarily include all studies that met our eligibility criteria and subsequently delineate a hierarchical order of preference for merging the outcomes. With this approach, we hope to compile the data in a way that is both comprehensive and as specific as possible.

Another challenge in evaluating MRS studies concerns an early methodological issue. As it is commonly true for many neuroimaging works, the sample size available is typically small, which brings an extra dilemma into question, as false positive and false negative results are more likely to align, creating a troublesome picture of the variables being studied. Given this framework, a comprehensive portrayal of state-of-the-art MRS knowledge for any given disease often proves to be problematic. In this protocol overview, we presented a model of summarization for MRS data from the prototypical case of ADHD. By following this protocol, we expect to be able to successfully group the data available on MRS metabolites and ADHD in order to achieve unified results for different brain regions in the form of multiple meta-analyses, despite the great diversity of methodological approaches among studies. Additionally, we hope to assess the quality of the MRS case-control studies produced and, as multiple studies align, to indirectly evaluate the reliability of the technique as designed thus far.

### Limitations

Whereas this approach represents our best efforts on unifying MRS data from different studies, an important drawback of this design must be mentioned. When merging mean results obtained for metabolites, we are, so to say, considering that all works involved are targeting specifically the same areas, while we know that this is arguably never the case, even when the same equipment and personnel are involved. On a larger scale, we are likely to have to occasionally merge data from different voxels on the basis of neuroanatomy and neurofunctional proximity, as described on our methods. Regarding this limitation, we believe it is important to acknowledge that MRS is an estimation of brain metabolite concentrations within a given volume of interest rather than a fine magnifier of brain neurochemistry, its contribution being more on a macroscopic scale. Even so, when present, this problem should be addressed and examined considering its own significance. Illustrations of the brain indicating the disparities and overlap among studies, as in a sort of neural Venn diagram, will hopefully help to discuss the reliability of each result.

Another limitation of our protocol refers to our broad inclusion criteria, which will include different diagnostic methods and mostly rely on the judgment of the authors of each study, probably blending different diagnostic rationales and different presentations of the same disorder. Nevertheless, at present, this seems to be the only way to pool a reasonable amount of data for our purposes. Also, our primary goal is to investigate MRS metabolites in the general diagnostic standard currently available. As we are still working on a symptom-based approach for diagnosis in psychiatry, biological parameters still have to derive from non-biological clustering, and only subsequent analyses will be able to provide data for different methods in the future.

## Conclusion

In summary, we believe that our protocol of systematic review and meta-analysis might prove helpful in broadening the use of MRS data. MRS is a potentially fruitful approach for neuroscientific endeavor, and the body of knowledge already generated from it can be used to draw a unified understanding of metabolite concentrations for specific disorders. The heterogeneity in brain areas studied and the need for broad inclusion criteria due to the relatively low number of studies available would probably be the main limitations of our approach. Even so, we hope that our methodology will support us to gather multiple estimates of how much of a substance is present in living neural tissue, opening a vast window of investigation for which the full employment remains to be explored. Therefore, following this protocol will potentially allow us to use the MRS data produced so far to enlighten our current understanding of either ADHD or any other brain disorder. Last, in succeeding in our purpose, we would conceivably provide a background capable of encouraging more systematic approaches to MRS studies.

**Figure 1:**
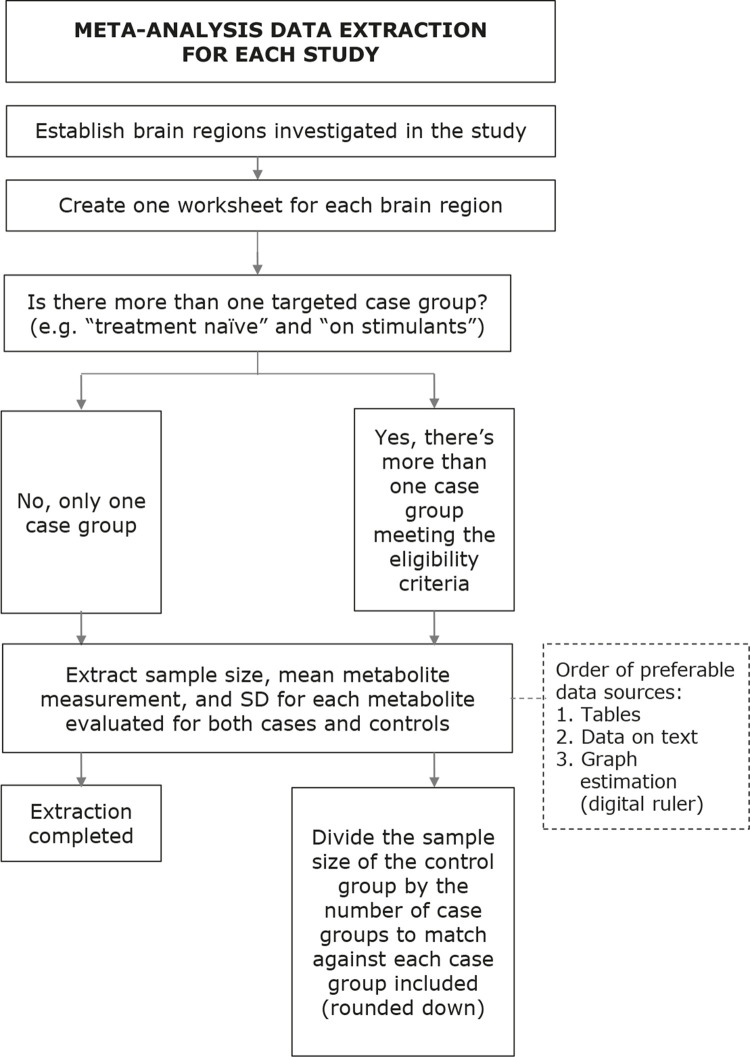
Meta-analysis data extraction. SD = standard deviation.

**Figure 2:**
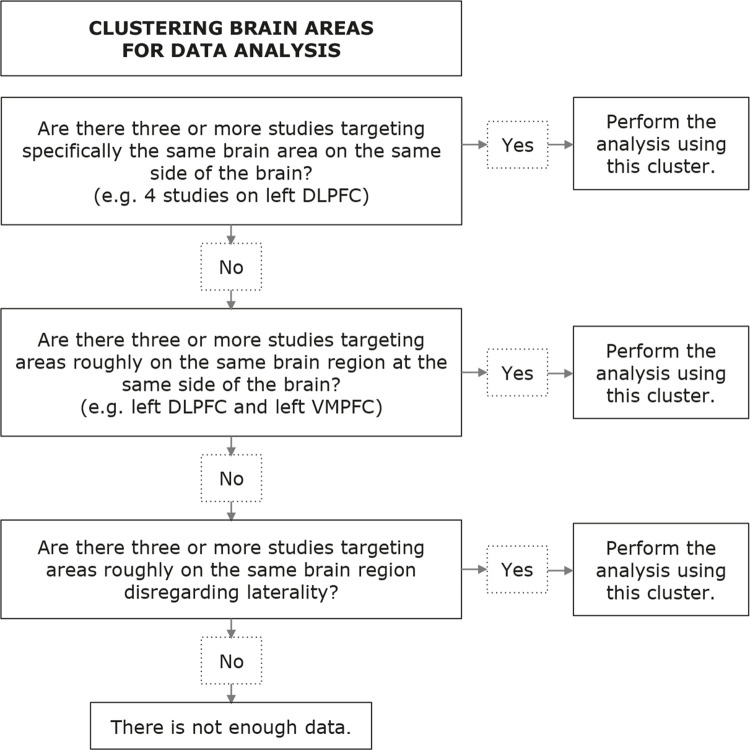
Clustering brain areas. DLPFC = dorsolateral prefrontal cortex; VMPFC = ventromedial prefrontal cortex.
